# Sexual selection drives weak positive selection in protamine genes and high promoter divergence, enhancing sperm competitiveness

**DOI:** 10.1098/rspb.2009.0257

**Published:** 2009-04-01

**Authors:** Juan Martin-Coello, Hernán Dopazo, Leonardo Arbiza, Juan Ausió, Eduardo R.S. Roldan, Montserrat Gomendio

**Affiliations:** 1 Reproductive Ecology and Biology Group, Museo Nacional de Ciencias Naturales (CSIC)c/José Gutiérrez Abascal 2, 28006 Madrid, Spain; 2 Bioinformatics and Genomics Department, Centro de Investigación Príncipe Felipe (CIPF)Valencia 46013, Spain; 3 Department of Biochemistry and Microbiology, University of VictoriaVictoria, British Columbia V8W 3P6, Canada; 4 Department of Veterinary Basic Sciences, The Royal Veterinary CollegeLondon NW1 0TU, UK; 5 Department of Zoology, University of CambridgeDowning Street, Cambridge CB2 3EJ, UK

**Keywords:** protamine genes, gene expression, sexual selection, sperm competition, spermatozoa, speciation

## Abstract

Phenotypic adaptations may be the result of changes in gene structure or gene regulation, but little is known about the evolution of gene expression. In addition, it is unclear whether the same selective forces may operate at both levels simultaneously. Reproductive proteins evolve rapidly, but the underlying selective forces promoting such rapid changes are still a matter of debate. In particular, the role of sexual selection in driving positive selection among reproductive proteins remains controversial, whereas its potential influence on changes in promoter regions has not been explored. Protamines are responsible for maintaining DNA in a compacted form in chromosomes in sperm and the available evidence suggests that they evolve rapidly. Because protamines condense DNA within the sperm nucleus, they influence sperm head shape. Here, we examine the influence of sperm competition upon protamine 1 and protamine 2 genes and their promoters, by comparing closely related species of *Mus* that differ in relative testes size, a reliable indicator of levels of sperm competition. We find evidence of positive selection in the protamine 2 gene in the species with the highest inferred levels of sperm competition. In addition, sperm competition levels across all species are strongly associated with high divergence in protamine 2 promoters that, in turn, are associated with sperm swimming speed. We suggest that changes in protamine 2 promoters are likely to enhance sperm swimming speed by making sperm heads more hydrodynamic. Such phenotypic changes are adaptive because sperm swimming speed may be a major determinant of fertilization success under sperm competition. Thus, when species have diverged recently, few changes in gene-coding sequences are found, while high divergence in promoters seems to be associated with the intensity of sexual selection.

## 1. Introduction

Phenotypic evolution may occur through changes in gene-coding sequences (CDSs) or regulatory regions, but the relative contribution of the two is a matter of debate. Some authors argue that regulatory mutations may have a predominant role in phenotypic evolution, mainly because the modular nature of regulatory regions largely frees them from deleterious pleiotropic effects, thus allowing selection to operate more efficiently by minimizing functional trade-offs (Carroll 2005; Wray 2007). The main argument against this view is that there is still little empirical evidence to support the role of regulatory mutations on phenotypic evolution, while there is ample evidence that structural mutations do play an important role (Hoekstra & Coyne 2007). However, this may be owing to the fact that fewer studies have examined the role of changes in regulatory regions, which is partly because, unlike coding regions, regulatory sequences are difficult to identify (Gilad *et al*. 2006). Several lines of evidence suggest that gene regulation may play an important role in evolutionary change, although this evidence is not conclusive. First, genomic studies have found interspecific divergence in gene expression, but they have not been able to link this divergence to phenotypic effects (Pollard *et al*. 2006). Second, single-locus studies have compared differences in phenotype among species with the pattern of expression of a single gene thought to influence that phenotype (Beldade *et al*. 2002; Reed & Serfas 2004; Abshanov *et al*. 2006). Because such comparisons commonly involve distantly related species, there are often differences in the coding regions as well as in the regulatory regions, making it difficult to rule out the effect of structural mutations. Third, there is evidence that changes in regulatory elements are linked to adaptive traits (Sucena & Stern 2000; Shapiro *et al*. 2004; Prud'homme *et al*. 2006), but individual mutations in regulatory elements have not been identified and all the cases involve trait loss.

Some studies have found that the rate of protein divergence between species is not correlated with the rate of expression divergence (Larracuente *et al*. 2008), although other studies have reached different conclusions (Khaitovich *et al*. 2005). This suggests that in some genes, positive selection may act at the level of protein sequence, but not at the level of gene expression, and *vice versa* in other genes. Genes with male-biased expression consistently show the greatest divergence between species at both sequence and expression levels (Ellegren & Parsch 2007). Most studies have focused on differences between species in genes expressed in male reproductive tissues, showing evidence of positive selection when distantly related species are compared (Swanson & Vacquier 2002; Torgerson *et al*. 2002). The fact that these reproductive genes are expressed in the male germ line and accessory glands suggests that such rapid changes may have been favoured by sperm competition. Despite the popularity of this hypothesis, there is limited supporting evidence. An association between the rate of evolution of reproductive genes and levels of sperm competition has been reported for semenogelin II, a structural component of semen coagulum in primates (Dorus *et al*. 2004; but see Hurle *et al*. 2007), and *Svs*2, which encodes the major component of the rodent copulatory plug (Ramm *et al*. 2008). In addition, the rate of evolution of sperm zonadhesin has been found to be inversely related to the degree of sexual dimorphism in primates, which the authors assume to be related to levels of sperm competition (Herlyn & Zischler 2007). However, no study so far has shown how differences in gene and protein sequences lead to differences in ejaculate competitiveness upon which selection could plausibly operate. The evolution of expression of reproductive genes has received much less attention, and no study so far has examined whether sexual selection does play a role in the evolution of the expression of reproductive genes.

Among mammals, evidence of positive selection in reproductive proteins has often been found when comparing distantly related species, but studies on closely related species have generated conflicting results (Jansa *et al*. 2003; Turner & Hoekstra 2006). A study on muroid rodents has found evidence of positive selection in four out of seven reproductive genes examined, but evidence suggesting that sexual selection favours adaptive evolution has been found for only one reproductive gene (Ramm *et al*. 2008). Studies on groups of species where reproductive isolation has evolved recently are needed to identify the first changes promoting divergence between species, and to understand the adaptive significance of such early changes. Comparing divergence in CDSs and promoters during the incipient stages of speciation will allow us to find out whether sexual selection operates more efficiently at one level or the other. The fact that studies on closely related species either do not find evidence of positive selection in reproductive genes or fail to establish a link between evolutionary rates and levels of sexual selection for most genes suggests that, during the early stages of divergence, sperm competition may enhance sperm competitiveness largely by influencing gene expression.

Protamines have been found to be among the fastest-evolving reproductive proteins in mammals (Wyckoff *et al*. 2000; Torgerson *et al*. 2002; Ramm *et al*. 2008), but the selective forces driving this extremely rapid rate are a matter of debate. Wyckoff *et al*. (2000) found evidence of more diverged sequences among humans and chimpanzees than gorillas, and argued that sperm competition may play a role under the assumption that protamines influence sperm morphology and male fertilizing ability. However, this study has been criticized for two main reasons: it fails to provide a link between the processes that protamines regulate and sperm competition mechanisms, and it focuses on gene sequences whereas it is the levels of protamine expression that have been found to be related to male fertility (Clark & Civetta 2000). An alternative hypothesis suggests that protamines are under purifying selection (Rooney *et al*. 2000).

Protamines are the most abundant sperm nuclear basic proteins in many species and are responsible for maintaining DNA in a compacted form in chromosomes in sperm (Oliva 2006). In mammals, there are two types of protamine: protamine 1 (*Prm 1*) and protamine 2 (*Prm 2*). *Prm 1* is present in all species of vertebrates studied, but *Prm 2* is present only in some species, including rodents, ungulates and primates (Corzett *et al*. 2002), which could indicate a more basic and conserved function for *Prm 1* and an accessory function for *Prm 2* in some species.

Here, we test whether sexual selection drives rapid evolution in *Prm 1* and *Prm 2* genes and their promoters by comparing 10 closely related species of *Mus* that differ in the levels of sperm competition. Previous work has shown that sperm competition has had a profound influence on the evolution of reproductive traits in this group of species, influencing not only the rate of sperm production but also the proportion of sperm ready to fertilize and their sensitivity to ovum signals (Gomendio *et al*. 2006). In addition, we have been able to show recently that, in this group of species, sperm competition plays an important role in favouring rapid changes both in sperm (increased sperm competitiveness) and ova (increased ovum defensiveness), which lead to incipient reproductive barriers (Martin-Coello *et al*. 2009).

## 2. Material and Methods

### (a) Collection of testes

The study includes males from 10 species of *Mus*: *Mus cookii*, *Mus famulus*, *Mus macedonicus*, *Mus musculus bactrianus*, *Mus musculus castaneus*, *Mus musculus domesticus*, *Mus musculus musculus*, *Mus pahari*, *Mus spicilegus* and *Mus spretus*. *Apodemus sylvaticus* was used as an outgroup. Previous studies have shown that, despite being closely related, interspecific differences in levels of sperm competition are associated with increases in sperm competitiveness and ovum defensiveness, which lead to asymmetric reproductive barriers (Gomendio *et al*. 2006; Martin-Coello *et al*. 2009).

Males of these *Mus* species were purchased from the Institut des Sciences de l'Evolution, CNRS-Université Montpellier 2, France. *Apodemus sylvaticus* males were caught in the wild (Sierra de Guadarrama, Madrid, Spain). Animals were kept and bred at the MNCN animal facility under 14 L : 10 D, and 22°C. All the males were kept in individual cages after weaning and allowed free access to food and water.

Males (*n*=5 for each species) were sacrificed by cervical dislocation and weighed. Testes were removed, weighed, frozen by direct immersion in liquid nitrogen and stored at −70°C. Relative testes size was calculated according to the rodent regression equation of Kenagy & Trombulak (1986), i.e. as observed mass of both testes/expected mass, where expected mass=0.031×body mass^0.77^. For each species, relative testes size was used as a reliable indicator of sperm competition levels.

### (b) Sperm parameters

Spermatozoa were collected from epididymides and vasa deferentia by allowing sperm to swim out for 7 min into a HEPES-buffered modified Tyrode's medium (mT-H; Shi & Roldan 1995).

Sperm morphology was assessed by staining sperm smears with Giemsa (Watson 1975; Tamuli & Watson 1994). Smears were air-dried and fixed in 4 per cent formaldehyde in tartrate–phosphate buffer (TPB). The smears were washed with water for 10 min, then submerged for 60 min in a Giemsa solution (4.5 ml Giemsa stock solution, 3 ml buffer TPB and 32.5 ml distilled water). The stained smears were washed with distilled water, dried and mounted using DePeX (BDH, Madrid, Spain). Sperm abnormalities were classified depending on whether they affected the head, midpiece or rest of the flagellum.

Sperm dimensions were obtained in Giemsa-stained cells. Images were captured using a microscope (Nikon Labophot 2) with a 40× objective under bright field and a monochrome charge-coupled device video camera (Sony SSC-M370CE). Images were digitized and analysed in an IBM-compatible computer using Visilog software (Visilog v. 4.1.3 Rev 6, Noesis, Vélizy, France).

Objective measures of sperm motility were recorded in spermatozoa suspended in mT-H medium, using a computer-aided sperm analyser (Hobson Sperm Tracker, Sheffield, UK). Assessments were made within 5 min of sperm collection for all species. A total of five descriptors of sperm motility were scored by analysing a minimum of 100 tracks per sample: (i) curvilinear velocity (VCL), velocity over total distance moved, including all deviations of sperm head movement; (ii) straight-line velocity (VSL), velocity calculated using the straight-line distance between the beginning and end of the sperm track; (iii) average path velocity (VAP), velocity over a calculated, smoothed path, which is a shorter distance than that used for calculating VCL; (iv) amplitude of lateral head displacement, the mean value of the extreme side-to-side movement of the sperm head in each beat cycle; and (v) linearity (LIN), the ratio (as a percentage) of the distances of straight-line track length/actual track length (this value is 100% for a completely linear track).

### (c) DNA isolation (phenol–chloroform extraction)

DNA extraction was performed by using a modified phenol–chloroform–isoamylalcohol method (Sambrook *et al*. 1989). Testes were ground in a mortar with liquid nitrogen until a fine powder was obtained. The powder was thawed in 10 ml of extraction buffer (10 mM Tris–HCl (pH 8.0), 0.1 M EDTA, 20 mg RNAse, 0.6% sodium dodecyl sulphate). The sample was mixed by vortexing and incubating for 60 min at 37°C, then 50 μl of stock solution of 20 mg proteinase K ml^−1^ were added. The sample was mixed by gentle swirling and incubated at 50°C for 3 hours with gentle swirling. The sample was then cooled to room temperature and an equal volume of phenol–chloroform (25 : 24) was added. The sample was then incubated overnight at 4°C.

The following day the sample was centrifuged at 5000 r.p.m. in a microfuge for 10 min at 4°C in order to separate the aqueous and organic phases. The supernatant was transferred to a clean tube to which an equal volume of phenol–chloroform (25 : 24) was added. The sample was incubated for 10 min at 4°C, and then centrifuged at 5000 r.p.m. for 10 min at 4°C to separate the aqueous and organic phases. This step was repeated twice. After the last centrifugation, the supernatant was transferred to another tube and an equal volume of phenol–chloroform–isoamylalcohol (25 : 24 : 1) was added, followed by incubation for 10 min at 4°C. The sample was centrifuged again for 10 min at 4°C, the supernatant transferred to another tube, and 0.2 volumes of 10 M NH_4_OAc and two volumes of 95 per cent ethanol were added.

A bent Pasteur pipette was used to allow the DNA to clump onto the pipette, which was then dipped twice in a tube with 5 ml of 70 per cent ethanol. Collected DNA was transferred to a tube with 2 ml of TE (10 mM Tris–HCl (pH 8.0), 0.1 mM EDTA) and stored at 4°C.

### (d) PCR and cloning of PCR products

Genomic DNA extracted from testes was used as template in polymerase chain reactions (PCRs).

Primers used for protamine 1 (*Prm 1*) were

- forward: 5′-CTCCCGGCCAAGCCAGCACC-3′,
- reverse: 5′-GGACTTGCTATTCTGTGCAT-3′.

Primers used for protamine 2 (*Prm 2*) were

- forward: 5′-CCTCCTGATCTCCTGGCACC-3′,
- reverse: 5′-ATGGACAGGCCTGGGGAGGC-3′.

Primers used for protamine 1 promoter (*Prm 1* promoter) were

- forward: 5′-CTGCGGCAGCATCGGTATCT-3′,
- reverse: 5′-TCCTCAGGACATGGTGGGCC-3′.

Primers used for protamine 2 promoter (*Prm 2* promoter) were

- forward: 5′-ATTCGGTAGCGAACCATGGT-3′,
- reverse: 5′-AAGAGTTGCCTTGGTCACGT-3′.

Primers were designed using the *M. m. musculus* DNA sequences for *Prm 1* (GenBank accession number NM 013637) and *Prm 2* (GenBank accession number NM 008933).

PCRs contained PCR buffer (50 mM KCl, 10 mM Tris–HCl (pH 8.3), 1.5 mM MgCl_2_), 200 μM each of the four deoxynucleoside triphosphates, 2 U of Taq DNA polymerase, 0.5 mM of each primer and 100 ng of DNA.

Resulting PCR products were purified using Wizard PCR Preps DNA Purification System (Promega, Alcobendas, Spain). Purified PCR products were then inserted into pCR 2.1-TOPO vector (Invitrogen, Barcelona, Spain) following the manufacturer's instructions and transformed into TOP10 competent cells (Invitrogen). Transformed cells were plated on Luria–Bertani (LB)-agar medium containing ampicillin and 5-bromo-4-chloro-3-indolyl-β-d-galactopyranoside (X-Gal) for the selection of positive clones. Positive colonies were picked and used to inoculate in tubes containing LB medium plus ampicillin, which were incubated overnight in humidified containers at 37°C with shaking.

The following day plasmid was purified using Wizard PlusMinipreps DNA Purification System (Promega), followed by restriction digestion with ECO RI (Roche Diagnostics, Barcelona, Spain) in order to confirm positive clones; positive clones were finally sequenced.

Sequence alignments were performed using the distance-based program ClustalX v. 1.83 (Thompson *et al*. 1997) with default parameters.

### (e) Phylogenetic analyses

**Figure 1:**
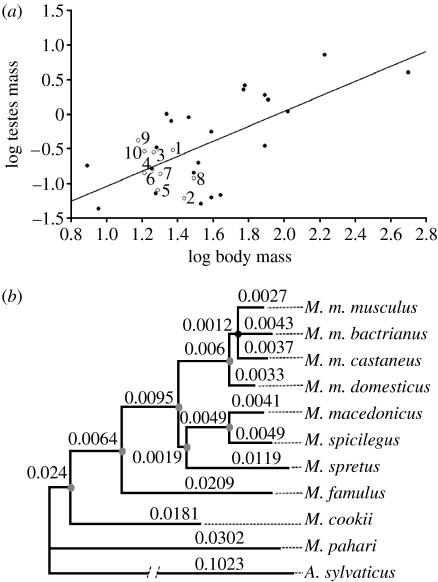
(*a*) Relationship between body weight and testes weight in murid rodents (*r*^2^=0.4463, *n*=32, *p*<0.0001). Open circles (our own data): 1, *M. cookii*; 2, *M. famulus*; 3, *M. macedonicus*; 4, *M. m. bactrianus*; 5, *M. m. castaneus*; 6, *M. m. domesticus*; 7, *M. m. musculus*; 8, *M. pahari*; 9, *M. spicilegus*; 10, *M. spretus*. Filled circles (data from Kenagy & Trombulak 1986): *Apodemus agrarius*, *Apodemus flavicollis*, *Apodemus microps*, *A. sylvaticus*, *Micromys minutus*, *Notomys alexis*, *Notomys cervinus*, *Notomys fuscus*, *Notomys mitchelli*, *Praomys natalensis*, *Pseudomys apodemoides*, *Pseudomys australis*, *Pseudomys delicatulus*, *Pseudomys desertor*, *Pseudomys gracilicaudatus*, *Pseudomys hermannsburgensis*, *Pseudomys nanus*, *Pseudomys novaehollandiae*, *Pseudomys shortridgei*, *Rattus exulans*, *Rattus norvegicus*, *Rattus rattus*. (*b*) Bayesian phylogenetic reconstruction of the 10 species of *Mus*. Note the low mean number of amino acid substitutions per site measured in the branches. Grey and black nodes represent clusters with 1.00 and 0.79 posterior probability values, respectively (table S1 in the electronic supplementary material).

A phylogenetic tree was constructed using sequences available in GenBank for the species used in this study (table S1 in the electronic supplementary material). CDSs were aligned using translated protein sequences as templates by means of Muscle (Edgar 2004) and default parameters. Phylogenetic testing of the best nucleotide substitution model was done using Modeltest program (Posada & Crandall 1998). The ungapped number of characters ranged from 11 038 to 13 073 for *M. m. domesticus*, *M. cookii*, *M. macedonicus*, *M. m. castaneus*, *M. m. musculus*, *M. spretus* and *M. spicilegus*, from 4894 to 8397 for *M. famulus*, *M. pahari* and *A. sylvaticus,* and were 1645 for *M. m. bactrianus*. Maximum-likelihood and Bayesian analyses were run in PhyML (Guindon & Gascuel 2003) and MrBayes (Ronquist & Huelsenbeck 2003) programs according to the best-fit model of DNA. Convergence of the four Markov chains was obtained in 1 000 000 generations, and 500 samples out of 1000 were used to summarize the posterior probability of all the trees. Note that the species with the lower number of ungapped characters produces a polytomy with the lowest posterior probability value (0.79) observed in the tree (figure 1*b*).

Comparative analyses by independent contrasts (CAIC, v. 2.6.9; Purvis & Rambaut 1995) were used to control for phylogenetic effects.

### (f) Positive Darwinian selection

Evolutionary rates at the codon level were computed using the codeml program from the PAML (v. 3.15) package (Yang 2007). Maximum-likelihood site models were fitted to protamine sequences using the PhyML tree topology deduced above. Likelihood ratio tests were computed between results of alternative models in order to infer events of positive selection. Rate of variation among sites was modelled using four categories, codon frequencies were estimated under the F3×4 model and the kappa (ts/tv) parameter was optimized in all of the ML site models. The site models computed were M1, M2, M7 and M8 (Yang *et al*. 2000).

### (g) Evolutionary parameters of promoters

Promoter sequences for both protamine 1 and protamine 2, as well as the more common transcription activation elements (TATA box, Y-box, Tet1, CAAT box, CRE and half CRE sequence; Mayr & Montminy 2001; Aoki & Carrell 2003), were also analysed in order to find out whether differences in the sequence of promoters and the position of transcription activation elements could influence protamine expression.

The evolutionary distances of *Prm 1* and *Prm 2* promoters were compared against the concatenated alignments of introns (INT) available for the genes *SmcX* Salivary Androgen binding protein gene, *zp3-2*, *zp3-3*, *SmcY* and *tcp1*. INT sequences were downloaded from GenBank. Orthologous promoter regions of protamine genes and concatenated sequences of INT were aligned using Muscle. After Modeltest optimization, promoters of protamine 1, protamine 2 and INT sequences fitted HKY+G, K80+G and TN93 DNA models, respectively. In order to compare evolutionary rates between promoters and INT sequences, we generated a distribution of branch lengths for each group of sequences based on bootstrap replications. PhyML program was run according to the best-fit model on each alignment using 10 000 replicates, assuming a constrained topology corresponding to the protein tree deduced in this study. *Apodemus sylvaticus* was excluded since no INT information was obtained from the GenBank database. Therefore, divergence was estimated as the branch length between the internal node joining *M. m. musculus* and *M. famulus*, and each of the descendent species of this node.

The mean branch length distribution values for *Prm 1* promoter, *Prm 2* promoter and the INT were compared and used to study the relationship between promoter evolution, relative testes size and sperm swimming velocity. Linear regressions were performed using the R statistics package (Ihaka & Gentleman 1996).

Promoter and gene sequences for both protamines of all species included in this study have been deposited in GenBank (accession numbers: FJ411373–FJ411394).

## 3. Results and discussion

### (a) Sequence analysis and phylogeny

We have examined a group of 10 species of *Mus* that, despite being closely related, differ in the levels of sperm competition. As shown in figure 1*a*, these species show clear differences in relative testes size, which is a reliable indicator of levels of sperm competition in most taxa. Thus, the 10 species cover the whole range from high to low sperm competition levels.

To perform comparative analyses, we used the available sequences for a variety of genes (table S1 in the electronic supplementary material) and constructed a phylogenetic tree derived from a gapped alignment of 15 415 characters for the 10 *Mus* taxa, using *A. sylvaticus* as an outgroup (figure 1*b*). Modeltest selected the TN93+G+I, with 70 per cent of invariant sites and rate heterogeneity α parameter value of 0.725. No differences in topologies were observed between the results of ML and Bayesian approaches (figure 1*b*). All the clades found the maximum posterior probability with the exception of the group clustering *M. m. musculus*, *M. m. bactrianus* and *M. m. castaneus* (*p*=0.79). In this case, we follow the analysis assuming the polytomy.

Protamine sequences were obtained and amino acid sequences deduced (figs S1 and S2 in the electronic supplementary material). Genetic divergence among sequences was low (see branch lengths of figure 1*b*, and figs S1 and S2 in the electronic supplementary material). Protamine 1 amino acid sequences are identical for all species of *Mus*, with two exceptions: *M. famulus* and *M. pahari* revealed two amino acid substitutions (fig. S1B in the electronic supplementary material). The amino acid sequences of protamine 2 reveal changes on four residues: 22, 80, 98 and 106. Ingroup and outgroup species differentiate at a single residue, 98. *Mus cookii* and *M. pahari* share the basal state condition at residue 80, while *M. spicilegus* shows a derived state at residue 22, and *M. spretus*, *M. famulus* and *M. spicilegus* show a common derived state at position 106 (fig. S2B in the electronic supplementary material).

### (b) Positive selection in protamine genes

**Figure 2:**
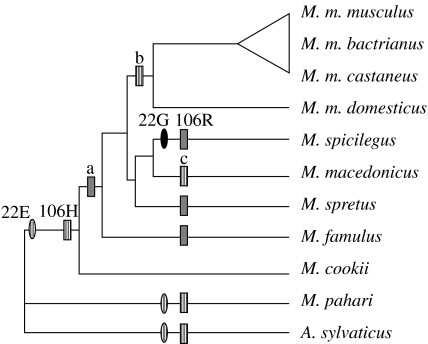
Main evolutionary events in protamine 2. Three independent events of amino acid change have occurred at residue 106 in protamine 2. The most frequent state and the basal condition of this site is histidine (H). The most parsimonious hypotheses of character evolution are (i) the independent transformation from 106H (hatched boxes) to 106R (grey boxes) in *M. spicilegus*, *M. spretus* and *M. famulus*, and (ii) its gain in the internal branch labelled ‘a’ and the posterior reversals in ‘b’ and ‘c’. An additional character state transformation has also occurred in site 22 of *M. spicilegus* where a glycine (G; black oval) is found instead of the basal condition of glutamic acid (E; hatched ovals). Site tests of positive selection found statistically significant evidence (*p*<0.05) of adaptive evolution associated with these changes (table 1). Note that two out of three species carrying derived states at residues 22 and 106 show the highest levels of sperm competition (figure 1*a*).

Using the phylogenetic tree of figure 1, we tested adaptive evolution on protamine genes by maximum-likelihood site test methods. Protamine 1 did not show evidence of adaptive evolution when using the site methods (table 1). By contrast, in protamine 2, positive selection was detected at residue 106 (*p*<0.01) when likelihoods of M2 and M8 positive selection models were compared with the corresponding values of M1 and M7 neutral models (table 1). In both cases, the likelihood ratio tests found statistically significant differences between them when considering the three possible topologies to arrange the trichotomy of figure 1*b*. Thus, in contrast to the results obtained for *Prm 1*, the statistical analyses performed on *Prm 2* suggest low values of gene divergence mainly modelled by a Darwinian process of adaptive evolution (figure 2).

### (c) Promoter evolution

**Figure 3:**
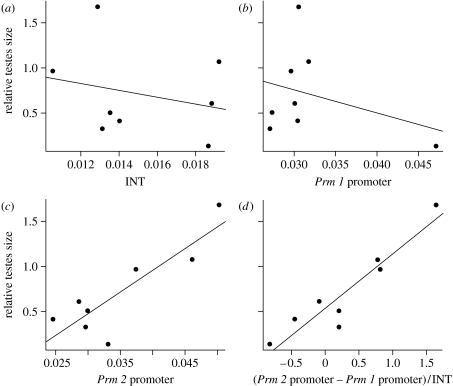
Promoter divergence and sexual selection. Results of the linear regression analyses between the genetic divergences of the regulatory elements of protamines against relative testes size. The divergence unit is the mean number of nucleotide substitution per site. Note that neither the evolution of (*a*) the intron sequences (INT, used as a neutral marker of evolution; *r*^2^=−0.096, *p*=0.5562) nor that of (*b*) the protamine 1 (*Prm 1*) promoter (*R*^2^=−0.043, *p*=0.4322) fits a linear regression model. Only when (*c*) the protamine 2 (*Prm 2*) promoter (*R*^2^=0.707, *p*=0.0055) divergence is considered alone or in combination with (*d*) other parameters of divergence (*Prm 2* promoter−*Prm 1* promoter)/INT; *R*^2^=0.86, *p*=0.0006) are significant relationships with relative testes size found. Genetic divergences of *Prm 1* promoter, *Prm 2* promoter and INT sequences were estimated as the mean distribution values of branch lengths computed after a 10 000 bootstrap-based analysis (see fig. S5 in the electronic supplementary material). *R*^2^ and *p* represent the fit and the statistical confidence of the linear regression models.

We also tested the possibility that sexual selection may enhance sperm competitiveness by promoting changes in gene regulation. We compared the degree of divergence in protamine 1 and protamine 2 promoters (*Prm 1* promoter and *Prm 2* promoter; figs S3 and S4 in the electronic supplementary material). Deletion and mutational analyses have revealed that changes in the sequence of *Prm 2* promoter may enhance *Prm 2* transcription by more than fivefold (Yiu & Hecht 1997). In addition, differences in the levels of expression of *Prm 2* between species have been associated with the differences in the efficiency of the promoters (Bunick *et al*. 1990). Both *Prm 1* and *Prm 2* promoters showed higher divergence rates than INT. In order to understand whether the genetic divergence of promoters of both protamine genes may be related to the intensity of sexual selection, the fit of a linear regression model of the mean evolutionary distances against relative testes size was evaluated. A highly significant relationship was found between *Prm 2* promoter divergence and relative testes size (*r*^2^=0.7065, *p*=0.0005)—independently or normalized in relation to *Prm 1* promoter and to the neutral divergence assumed for INT (figure 3; table 2). Neither *Prm 1* promoter nor the INT showed significant relationships with relative testes size. In general, *Prm 2* promoter accounts for most of the sequence variations observed on regulatory elements in these species (figs S3 and S4 in the electronic supplementary material). Remarkably, *M. spicilegus*, *M. spretus* and *M. macedonicus*, which in this order show the three highest absolute divergences in *Prm 2* promoter (fig. S5 in the electronic supplementary material), are also the species that show the highest relative testes size (figure 1*a*). Thus, species with high inferred levels of sperm competition show high divergence in *Prm 2* promoter.

Analysis of the most common transcription activation elements in the *Prm 1* promoter (table S2 in the electronic supplementary material) showed that TATA box, Y-box, Tet1 and CRE were in the same position in all species. The CRE half-sequence is also found at similar sites. We also found an insertion of 164 pb between positions −613 and −451 in *M. macedonicus* and *M. spicilegus*. This insertion is not a duplication of any other sequence of the promoter. In the case of the *Prm 2* promoter (table S3 in the electronic supplementary material), TATA box, Y-box and CAAT box were in the same position in all species; Tet1 was found in the same position in all species with the exception of *M. pahari*. Two different sequences of a CRE-like element were found in the *Prm 2* promoter and one of the sequences was found in *M. pahari*, while the rest of the species shared the same sequence. A CRE element was found at the same site in *M. pahari*, *M. famulus* and *M. cookii* (−184), but at a different site in the other species (−174). The CRE half-sequence is found at similar sites in all species. These findings suggest that differences in the sequence or position of the transcription factor binding sites discussed above are unlikely to account for the interspecific differences observed in the expression of protamines.

Sexual selection is thus associated with rapid changes in *Prm 2* promoters, which may modify the levels of gene expression. It is now well established that changes in protamine expression influence male fertility; in most cases, infertility is linked to changes in the expression of *Prm 2* (Carrell & Liu 2001; Aoki *et al*. 2005). In addition, the proportion between *Prm 1* and *Prm 2* levels is related to sperm viability, DNA integrity and fertilizing ability (Aoki *et al*. 2006). In mice, haploinsufficiency of protamines increases sperm morphological abnormalities and damage in sperm DNA, and decreases sperm motility (Cho *et al*. 2001). More specifically, *Prm 2*-deficient male mice have increased DNA damage, inefficient chromatin packaging, modified sperm heads and changes in the acrosome (Cho *et al*. 2003).

Thus, we tested the hypothesis that the rapid divergence observed in *Prm 2* promoters is associated with changes in sperm head dimensions and sperm swimming velocity. We analysed sperm morphology and sperm swimming velocity in these 10 *Mus* taxa. All analyses were corrected for phylogenetic effects using the phylogeny in figure 1*a*. After controlling for phylogenetic effects, relative testes size was found to be associated with head width (*n*=7 contrasts, *r*^2^=0.764, *p*=0.004), the proportion of sperm with head abnormalities (*n*=7 contrasts, *r*^2^=0.728, *p*=0.007) and sperm swimming velocity (VAP: *n*=7 contrasts, *r*^2^=0.688, *p*=0.01; VCL: *n*=7 contrasts, *r*^2^=0.548, *p*=0.03; VSL: *n*=7 contrasts, *r*^2^=0.645, *p*=0.01; LIN, *n*=7 contrasts, *r*^2^=0.631, *p*=0.01). Thus, species of rodents with inferred high levels of sperm competition have (i) a lower proportion of sperm with head abnormalities, and (ii) sperm with narrower sperm heads, which (iii) swim faster and follow straighter trajectories.

**Figure 4:**
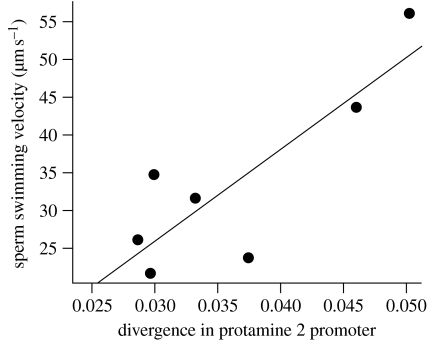
Relationship between mean values of divergence of the regulatory region controlling the expression of *Prm 2* and sperm swimming velocity (straight-line velocity: VSL, *R*^2^=0.659, *p*=0.02). The divergence unit is the mean number of nucleotide substitution per site.

Furthermore, we were able to establish a direct link between divergence in *Prm 2* promoters and sperm swimming velocity. Thus, the mean values of divergence of the regulatory region controlling the expression of *Prm 2* show a significant association with sperm swimming velocity (VSL, *r*^2^=0.659, *p*=0.02; figure 4), and a marginally non-significant association with LIN (*r*^2^=0.4448, *p*=0.061). Previous studies by our research group were able to show that faster-swimming sperm have more elongated and narrower heads (Malo *et al*. 2006). Thus, one possibility that deserves further study is that the association between sperm competition, divergence in *Prm 2* promoters and sperm swimming velocity is mediated by changes in the degree of condensation of DNA within the sperm head, which in turn may influence sperm head shape.

By contrast, no relationships were found between divergence in *Prm 1* promoters and sperm head morphology or dimensions, or sperm swimming velocity.

Our findings show that intense sexual selection is associated with rapid divergence in *Prm 2* promoters, which, in turn, correlates with faster sperm swimming speeds. A possible underlying mechanism could be that changes in *Prm 2* promoters lead to more efficient DNA condensation within the sperm head and this, in turn, results in more hydrodynamic sperm heads; but these causal links need to be tested. Previous studies have shown that males with faster-swimming sperm are more fertile (Malo *et al*. 2005), and that when rival ejaculates are placed in competition the fastest-swimming sperm win the race to fertilize the ova (Birkhead *et al*. 1999). Thus, the intensity of sexual selection is linked to changes in the regulation of *Prm 2*, which in turn are associated with increases in sperm swimming velocity, which may be a major determinant of fertilization success in sperm competition contexts.

In conclusion, sexual selection in a group of closely related species of rodents has no impact on *Prm 1* genes or its regulatory regions. By contrast, *Prm 2* shows low values of gene divergence but significant evidence that it is driven by adaptive evolution. Furthermore, sperm competition selects high divergence in the promoter region of *Prm 2*. These findings support the idea that the function of *Prm 1* is more conserved, while *Prm 2* may be more sensitive to the intensity of sexual selection. Our results also show that the marked divergence in protamine genes reported when distantly related species are compared is absent between closely related species.

Most changes in the sequence of *Prm 2* genes occur in the species with the highest inferred levels of sperm competition, while the relationship between relative testes size, divergence in *Prm 2* promoters and sperm swimming velocity is evident across the whole range of species included in this study. Thus, while evolutionary divergence in CDSs seems correlated with divergence in promoters to some extent, intense sexual selection is needed to detect changes in *Prm 2* genes, while divergence in promoters takes place at low and intermediate levels of sperm competition. This could indicate that the first steps in the evolutionary process towards reproductive isolation and species divergence may involve faster changes in regulatory elements, and therefore gene expression, than in gene sequences coding for proteins. Such high divergence in promoters could help to explain the rapid evolutionary responses to experimental manipulations of sperm competition intensity, which modify male reproductive traits (including sperm size) in just a few generations (LaMunyon & Ward 2002). These findings support the idea that, for short evolutionary time scales, CDSs would be expected to evolve more slowly than promoter regions.

By integrating several levels of analysis, we have been able to show how changes in behaviour (female sexual promiscuity) create new selective pressures (sperm competition), which favour minor changes in gene sequence (molecular adaptive evolution) and major changes in its regulation (promoter genetic divergence), which are associated with changes in both sperm design and sperm performance. We conclude that, in the incipient stages of speciation, sexual selection may favour more rapid divergence in the regulation of reproductive genes than in their structure, resulting in different evolutionary dynamics for coding and promoter sequences.

## Figures and Tables

**Table 1:** Positive selection on protamine genes. (Parameter estimation and likelihood scores under models of variable *ω* ratios among sites for protamine 1 (*Prm 1*) and protamine 2 (*Prm 2*). Protamine 1 does not show differences in likelihoods under alternative ML site models, suggesting the absence of positive selection in this protein. However, positive selection was detected on residue 106 of protamine 2 when likelihood ratio tests were applied to compare the alternative selection and nearly neutral models under two different models for the distribution of *ω* among sites (2Δ*l*_M1&M2_=12.60; 2Δ*l*_M7&M8_=12.62>*Χ*_(0.001,d.f.=1)_^2^=10.83). According to the Bayes empirical Bayes (BEB) analysis, residue 106 has a mean value of *ω* between 8.26 and 8.24 with a posterior probability of being positively selected between 0.994 and 0.998 in models M2 or M8, respectively. Residue 22 changing from glutamic acid (E) to glycine (G) in *M. spicilegus* does not reach the significance cut-off value (*P*_*ω*>1_=53.0%). The *ω* ratio is taken as the average over all sites in the alignments. PSS is the number of positively selected sites, inferred above a 50% posterior probability cut-off.)

	protein model	ln *L*	*ω*	parameter estimates	PSS
*Prm 1*	M1. nearly neutral	−79.8749	0.000	*p*_0_=1.00, (*p*_1_=0.00), (*ω*_0_=0.00), (*ω*_1_=1.00)	not allowed
	M2. positive selection	−79.8749	0.000	*p*_0_=1.00, *p*_1_=0.00, (*p*_2_=0.00), (*ω*_0_=0.00), (*ω*_1_=1.00), *ω*_2_=1.00	none
	M7. beta	−79.8749	0.000	*p*=0.0050, *q*=4.2971	not allowed
	M8. beta and *ω*	−79.8749	0.000	*p*_0_=1.00, *p*=0.005, *q*=0.1289, (*p*_1_=0.00), *ω*=1.00	none
*Prm 2*	M1. nearly neutral	−460.3668	0.272	*p*_0_=0.728, (*p*_1_=0.272), (*ω*_0_=0.00), (*ω*_1_=1.00)	not allowed
	M2. positive selection	−454.0647	1.438	*p*_0_=0.99, *p*_1_=0.00, (*p*_2_=0.01), (*ω*_0_=0.25), (*ω*_1_=1.00), *ω*_2_=118.95	106H: *P*_(ω>1,BEB)_=0.994
	M7. beta	−460.3764	0.250	*p*=0.0050, *q*=0.0193	not allowed
	M8. beta and *ω*	−454.0665	1.442	*p*_0_=0.99, *p*=9.6582, *q*=27.8447, (*p*_1_=0.01), *ω*=119.0678	22E: *P*_(ω>1,BEB)_=0.530, 106H: *P*_(ω>1,BEB)_=0.998

**Table 2:** Promoter evolution and relative testes size. (Statistical values of linear regression models between the genetic divergence of the regulatory elements of protamines and relative testes size. Note that, irrespective of the way in which the relative divergence of *Prm 2* promoter in relation to *Prm 1* promoter and/or INT sequences is analysed, all the variables considering the promoter region of *Prm 2* fit a linear regression model with statistical confidence (*p*<0.05). Genetic divergence of the *Prm 1* promoter, *Prm 2* promoter and INT sequences was estimated as the mean distribution value of branch lengths computed after a 10 000 bootstrap-based analysis (see fig. S3 in the electronic supplementary material). n.s., non-significant at 95% of statistical confidence. ^*^Significant at 95% of statistical confidence. ^**^Significant at 99% of statistical confidence. ^***^Significant at 99.9% of statistical confidence.)

variable	*R*^2^	*p*-value
INT	−0.0957	0.5662^n.s.^
*Prm 1* promoter	−0.0434	0.4322^n.s.^
*Prm 1* promoter/INT	−0.1631	0.8960^n.s.^
*Prm 2* promoter	0.7065	0.0055^**^
*Prm* 2 promoter/INT	0.5826	0.0168^*^
*Prm 2* promoter−*Prm 1* promoter	0.8091	0.0014^**^
(*Prm 2* promoter−*Prm 1* promoter)/INT	0.8599	0.0005^***^
*Prm 2* promoter/*Prm 1* promoter	0.8233	0.0011^**^
(*Prm 2* promoter−INT)/(*Prm 1* promoter−INT)	0.5619	0.0195^**^

## Supplementary Material

Martin-Coello_electronic supplementary material_figures Electronic supplementary material figures

Martin-Coello_electronic supplementary material_tables Electronic supplementary material tables
