# Supplementary material for: Sexual selection drives weak positive selection in protamine genes and high promoter divergence, enhancing sperm competitiveness
Source: Proc Biol Sci. 2009 Apr 1;276(1666):2427–36. doi: 10.1098/rspb.2009.0257 (PMC2690471; doi:10.1098/rspb.2009.0257)

FIGURES S1-S5

To the article:

Sexual selection drives weak positive selection in protamine genes and high promoter divergence, enhancing sperm competitiveness.

by

Juan Martin-Coello, Hernán Dopazo, Leonardo Arbiza, Juan Ausió, Eduardo R.S. Roldan, and Montserrat Gomendio.

Proc. R. Soc. B

Figure S1. Multiple alignment of protamine 1 sequences. (A) DNA and (B) protein sequences of 10 rodent species. Note that most of the coding variation in protamine 1 sequence corresponds with synonymous substitutions in the protein. Only 3 residues (at position 32, 36 and 51) differenciates *Apodemus sylvaticus* (outgroup) with the species of the ingroup

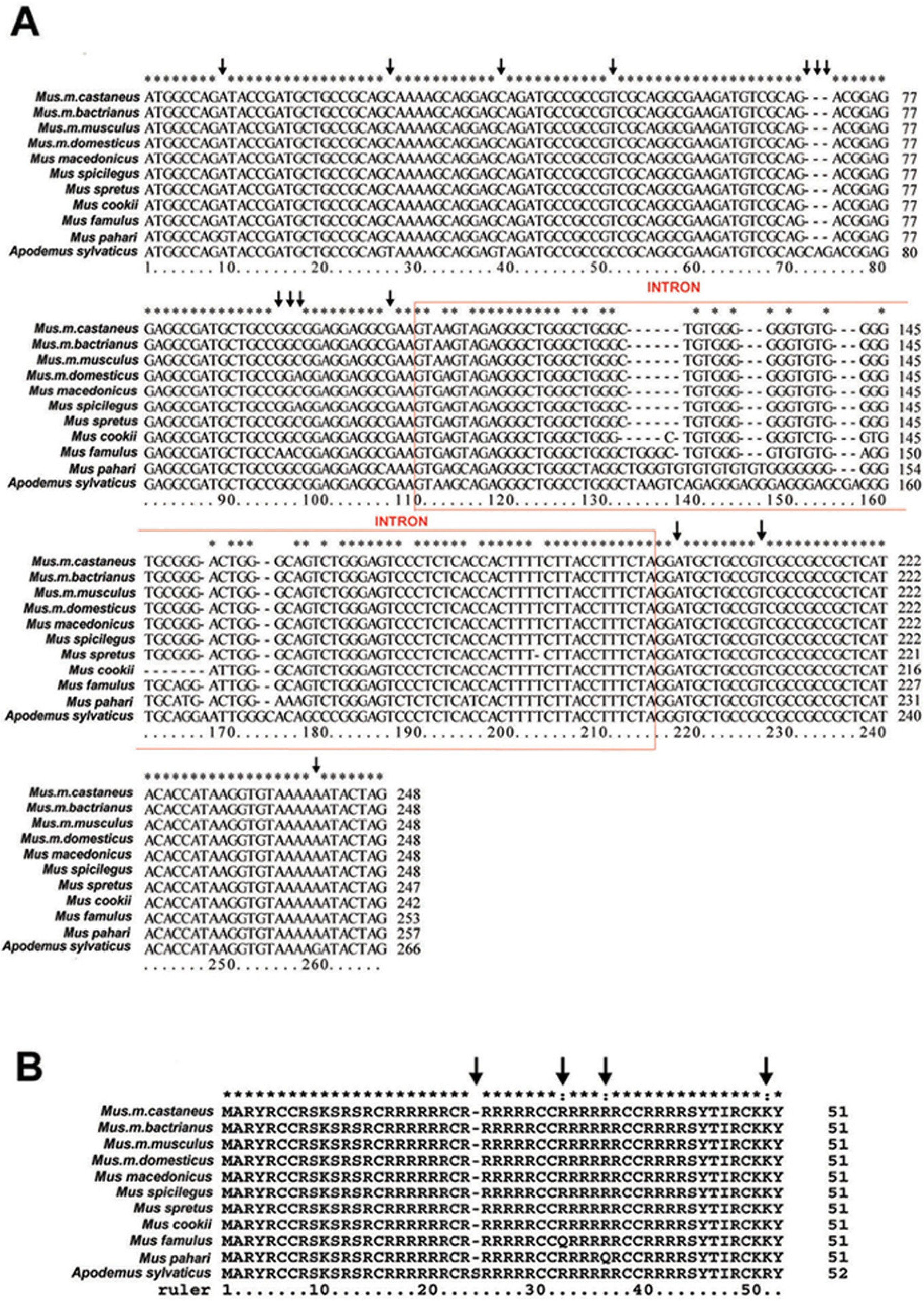

Figure S2. Multiple alignment of protamine 2 sequences. (A) DNA and (B) protein sequences of the 10 rodent species. Sequence alignments show non-synonymous variation at residues 22, 80 and 106. Non-synonymous variation at codon 98 differenciates *Apodemus sylvaticus* (outgroup) from all the (ingroup) species of *Mus*.

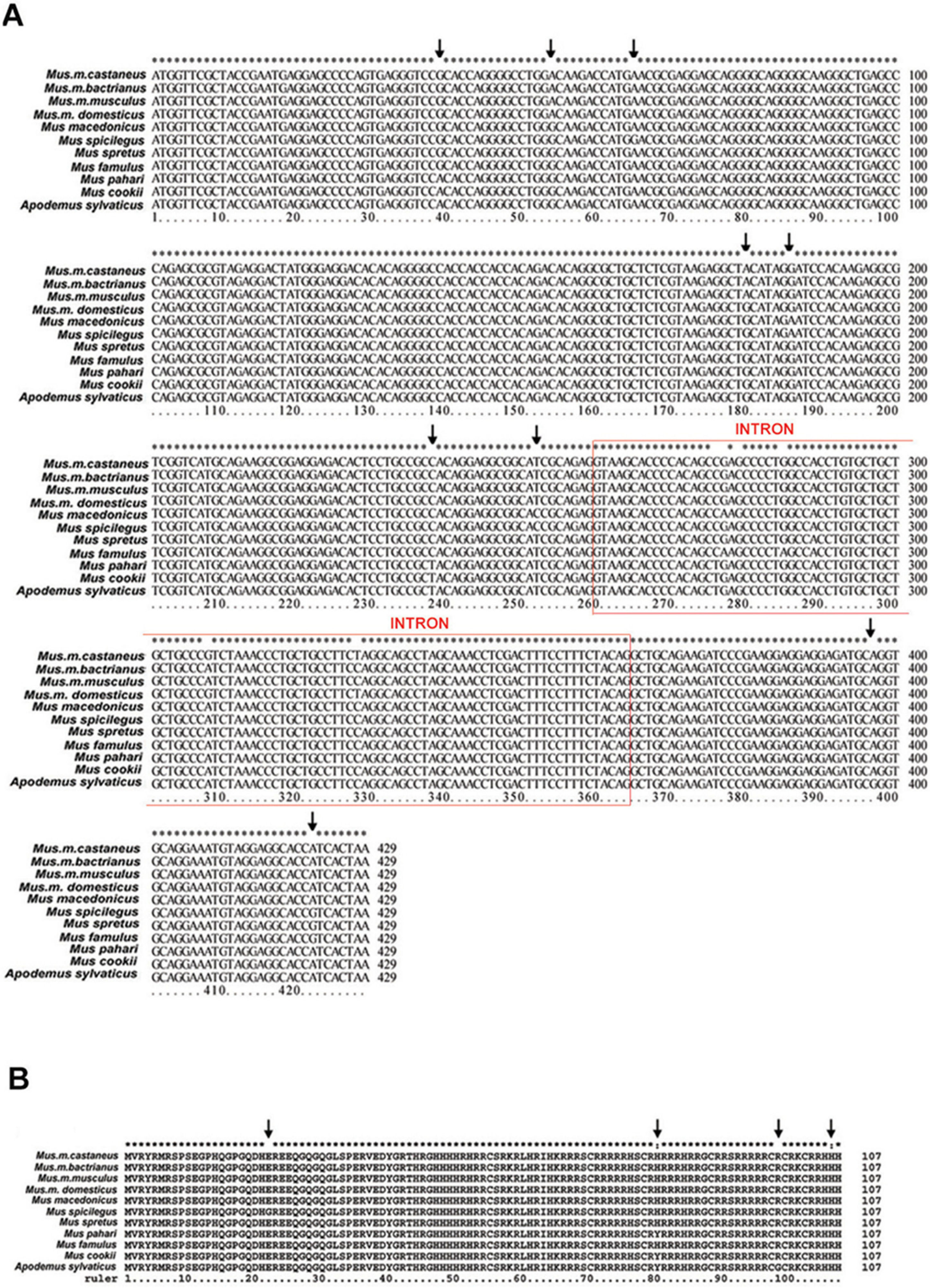

Figure S3. Multiple alignment of protamine 1 promoter sequences from 10 species of *Mus*.

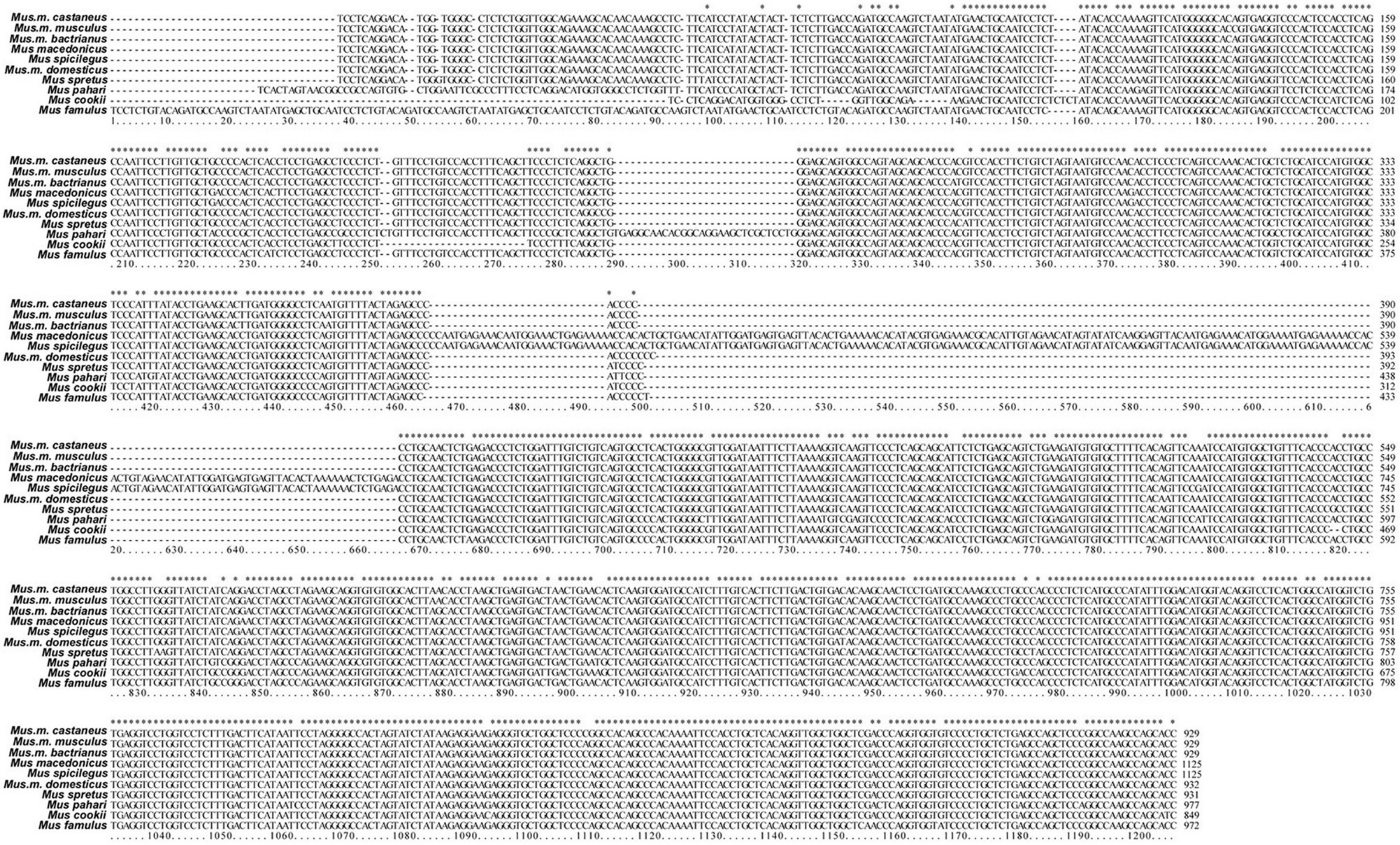

Figure S4. Multiple alignment of protamine 2 promoter from 10 species of *Mus*.

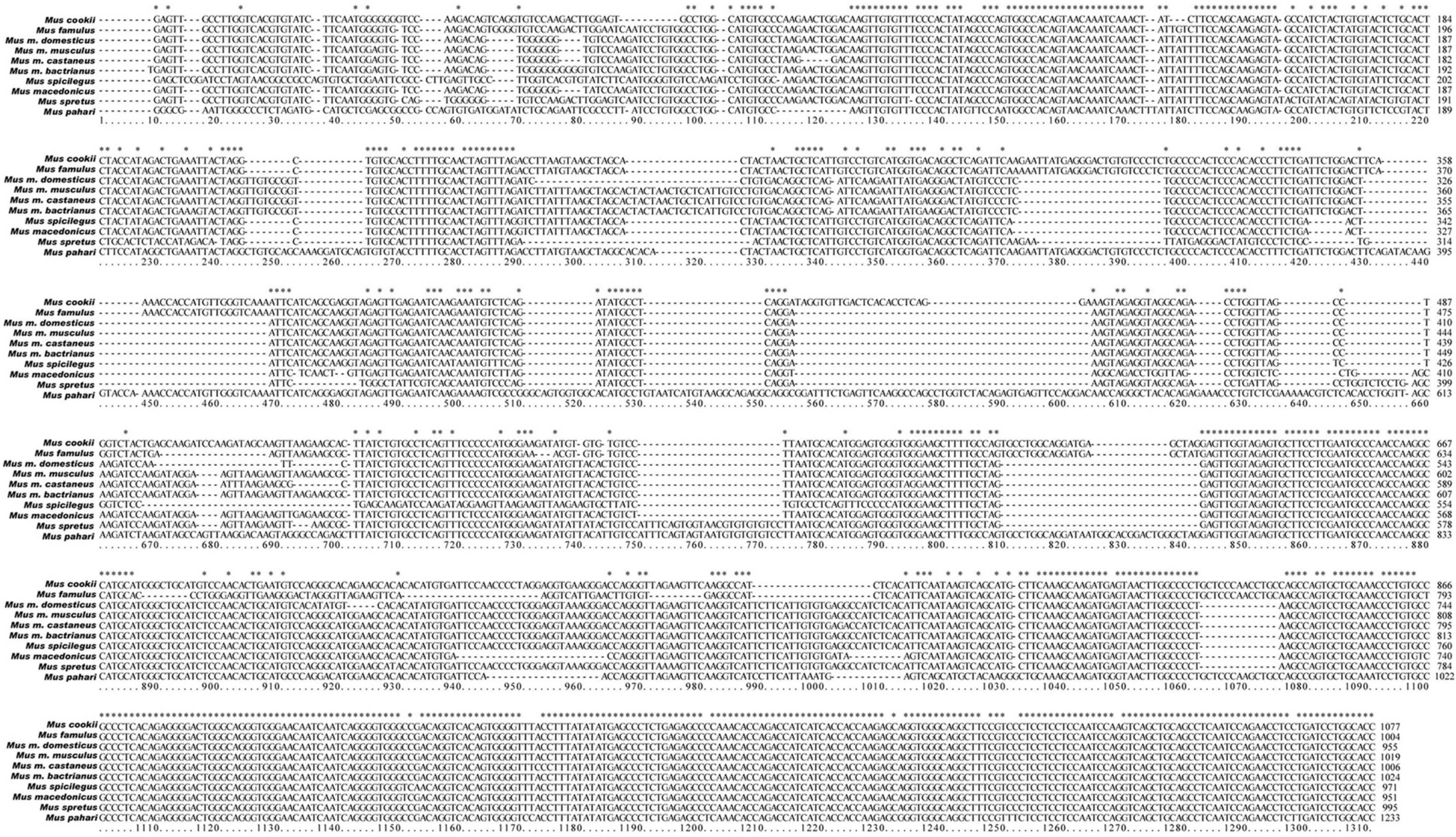

Figure S5. Divergence of promoter sequences. The probability density distribution of branch lengths from 10,000 replicates of protamine 1 promoter (pP1) and protamine 2 promoter (pP2) is shown together with that obtained from the intron sequences. The intron sequence was used as proxy of neutral divergence. *M. spicilegus*, *M. spretus* and *M. macedonicus*, which are the species with highest levels of sperm competition, show the highest lengths in pP2. *M. cookii* and *M. pahari* were excluded from the analysis since their branch lengths remain undefined in the unrooted intron tree built without the information of *Apodemus sylvaticus*.

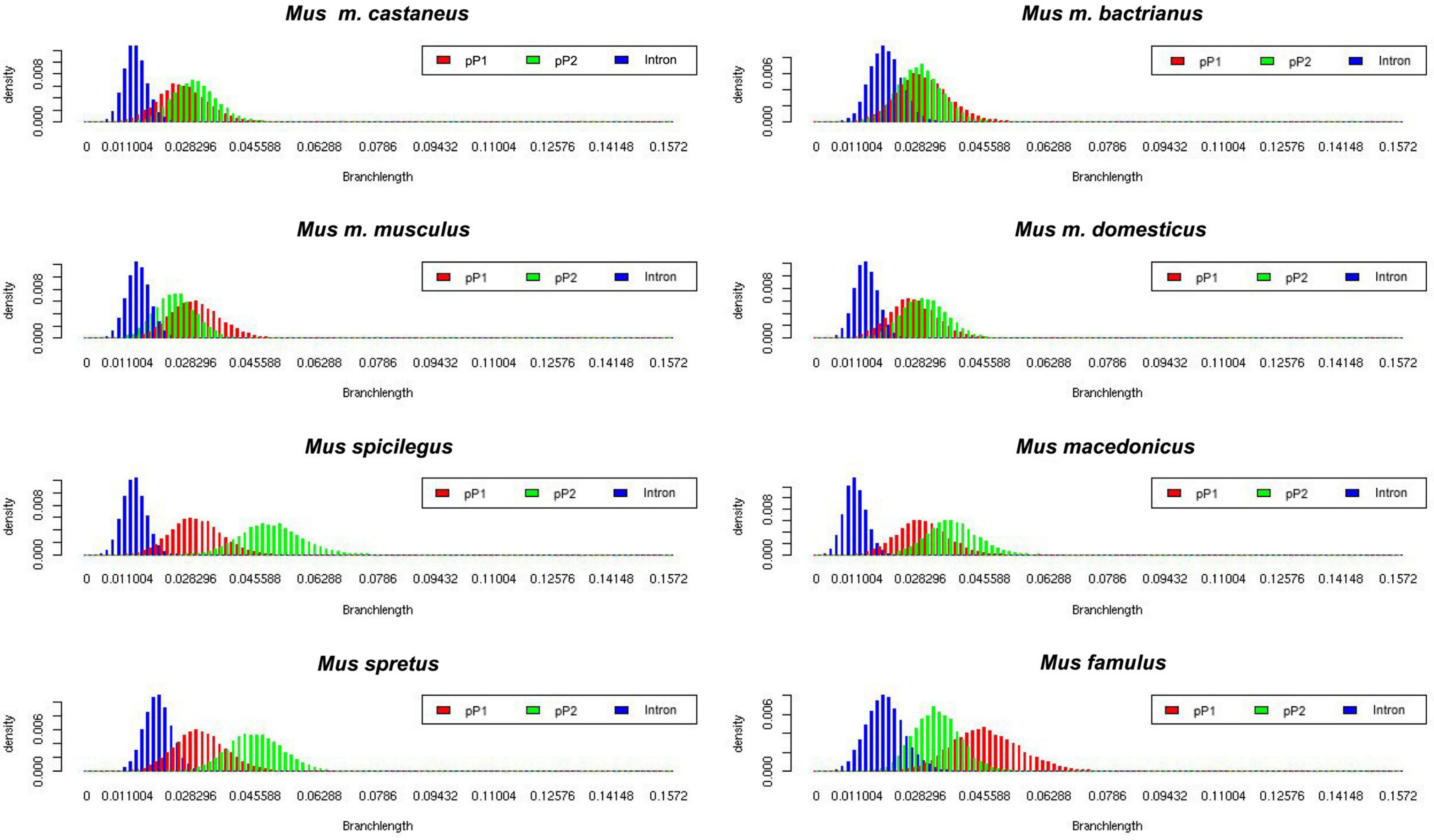

Supplement: Martin-Coello_electronic supplementary material_figures — Electronic supplementary material figures [file rspb20090257s17.pdf]
