# Supplementary material for: Sexual selection drives weak positive selection in protamine genes and high promoter divergence, enhancing sperm competitiveness
Source: Proc Biol Sci. 2009 Apr 1;276(1666):2427–36. doi: 10.1098/rspb.2009.0257 (PMC2690471; doi:10.1098/rspb.2009.0257)
Supplement: Martin-Coello_electronic supplementary material_tables — Electronic supplementary material tables [file rspb20090257s18.pdf]

# TABLES S1-S3

To the article:

Sexual selection drives weak positive selection in protamine genes and high promoter divergence, enhancing sperm competitiveness.

by

Juan Martin-Coello, Hernán Dopazo, Leonardo Arbiza, Juan Ausió, Eduardo R.S. Roldan, and Montserrat Gomendio.

*Proc. R. Soc. B*

Table S1. Genes used for phylogenetic analysis. GenBank accession numbers of genes from *Mus m. bactrianus* (M ba), *Mus m. castaneus* (M ca), *Mus cookii* (M co), *Mus m. domesticus* (M do), *Mus famulus* (M fa), *Mus macedonicus* (M ma), *Mus m. musculus* (M mu), *Mus pahari* (M pa), *Mus spicilegus* (M spi), *Mus spretus* (M spr) and *Apodemus sylvaticus* (A syl), used for the phylogenetic analysis.

| | M ba | M ca | M co | M do | M fa | M ma | M mu | M pa | M spi | M spr | A syl |
|---|---|---|---|---|---|---|---|---|---|---|---|
| **Interphotore-ceptor retinoid binding protein** | | AB125807 | AB125802 | | AJ698884 | AB125805 | AB033711 | AB096855 | AJ698882 | AB033708 | AB032863 |
| **Smc Y protein** | | AY260482 | AY260486 | AY260487 | | AY260490 | AY260481 | AY260488 | AY260483 | AY260485 | |
| **Smc Y protein** | | AY260494 | AY260498 | AY260499 | | AY260503 | AY260493 | AY260500 | AY260495 | AY260497 | |
| **CATSPER1** | | DQ021487 | DQ021498 | DQ021486 | | DQ021496 | DQ021492 | DQ021500 | DQ021494 | DQ021493 | |
| **X-linked zinc finger protein** | | AY160025 | AY160016 | AY160021 | | AY160013 | AY160024 | | AY160014 | AY160015 | |
| **Autosomal zinc finger protein** | | AY160012 | AY160000 | AY160008 | | AY160003 | AY160011 | | AY160004 | AY160002 | |
| **Y-linked zinc finger protein** | | AAO21819 | AY159976 | AY159986 | | | AY159989 | | AY159980 | AY159978 | |
| **Rag 1** | | AB125832 | AB125828 | AB125833 | | AB125829 | AY215075 | AB125844 | AB125835 | AB125836 | AB164041 |
| **Salivary androgen-binding protein alpha sub unit** | | AF413619 | AF413625 | AF144714 | AF413623 | AF413622 | AF039064 | | AF413621 | AF413620 | AF445762 |
| **MHC class II H2-E alpha chain precursor** | U13649 | | U89284 | U13648 | | U89290 | U89287 | | U89291 | U89289 | |
| **GPR33** | AY490678 | AY490628 | | AY490635 | AY490634 | AY490627 | AY490639 | | AY490632 | AY490631 | AY490614 |
| **S12** | | AY057792 | X85946 | AB042432 | AJ279442 | AY057794 | AY057791 | X84383 | AY057795 | AY057796 | AJ311126 |
| **Sry** | | U70657 | L29549 | L29551 | | L29547 | U70654 | L29543 | L29550 | L29544 | |
| **Cyt b** | | AY057805 | AY05813 | AY057807 | AJ698872 | AY057808 | AY057804 | AY057814 | AY057809 | AY057810 | AF159395 |
| **Tcp1** | X61211 | X61219 | AY057822 | X61214 | | X61213 | X61217 | AY057821 | X61216 | X61215 | |
| **B2m** | M84364 | M84366 | L05581 | M84363 | | L05899 | AY057800 | L05579 | L05578 | M84362 | |
| **Zp3-1** | | AY057747 | AY057755 | AY057749 | | AY057750 | AY057746 | AY057756 | AY057751 | AY057752 | |
| **Zp3-2** | | AY057762 | AY057770 | AY057764 | | AY057765 | AY057761 | AY057771 | AY057766 | AY057767 | |
| **Zp3-3** | | AY057777 | AY057785 | AY057779 | | AY057780 | AY057776 | AY57786 | AY057781 | AY057782 | |

Table S2. Analysis of transcription activation elements in the protamine 1 promoter. Position and nucleotide sequence of the most common enhance transcription elements in the protamine 1 promoter sequenced from 10 species of *Mus* genus.

| Element | Sequence | Species | Position |
|---|---|---|---|
| **TATA box** | TATAAGA | all species | -31 |
| **Y-box** | TCCTCACTGGCCAC | all species | -105 |
| **Tet 1** | TGACTTCATAA | all species | -64 |
| **CAAT box** | not found | | |
| **CRE** | TGACTTCA | all species | -64 |
| **CRE half sequence** | TGAC | *M. cookii* | -144, -174, -180, -219 |
| | | *M. famulus* | -174, -180, -219, -223 |
| | | *M. macedonicus* | -174, -180, -223, -861, -962 |
| | | *M. m. bactrianus* | -174, -180, -223, -766 |
| | | *M. m. castaneus* | -174, -180, -223, -766 |
| | | *M. m. domesticus* | -180, -223, -766 |
| | | *M. m. musculus* | -174, -180, -223, -766 |
| | | *M. pahari* | -174, -180, -219, -223, -796 |
| | | *M. spicilegus* | -174, -180, -223, -861, -962 |
| | | *M. spretus* | -174, -180, -223, -767 |
| | TTCA | | |
| | | *M. cookii* | -296, -317, -322, -565, -602, -707 |
| | | *M. famulus* | -298, -317, -324, -567, -616, -711 |
| | | *M. macedonicus* | -298, -317, -324, -762, -811, -906, -977 |
| | | *M. m. bactrianus* | -298, -317, -324, -615, -710, -781 |
| | | *M. m. castaneus* | -298, -317, -324, -615, -710, -781 |
| | | *M. m. domesticus* | -298, -317, -324, -618, -713, -784 |
| | | *M. m. musculus* | -298, -317, -324, -615, -710, -78 |
| | | *M. pahari* | -298, -324, -567, -646, -743, -814 |
| | | *M. spicilegus* | -298, -324, -811, -906, -977 |
| | | *M. spretus* | -298, -317, -324, -567, -616, -711 |

Table S3. Analysis of transcription activation elements in the protamine 2 promoter. Position and nucleotide sequence of the most common enhance transcription elements in the protamine 2 promoter sequenced from 10 species.

| Element | Sequence | Species | Position |
|---|---|---|---|
| **TATA box** | TATATA | all species | -36 |
| **Y-box** | ACAATCAATCAGG | all species | -83 |
| **Tet 1** | CGCCCTCACAG | *M. cookii*<br>*M. macedonicus*<br>*M. pahari*<br>*M. m. bactrianus*<br>*M. m. castaneus*<br>*M. m. domesticus*<br>*M. m. musculus*<br>*M. spicilegus*<br>*M. spretus* | -115 |
| | TGCCCTCACAG | *M. famulus* | -115 |
| **CAAT box** | CAAT | all species | -78 |
| **CRE** | GGGCTGCA | *M. pahari* | -184 |
| | ATGCTTCA | *M. cookii*<br>*M. famulus* | -184 |
| | | *M. macedonicus*<br>*M. m. bactrianus*<br>*M. m. castaneus*<br>*M. m. domesticus*<br>*M. m. musculus*<br>*M. spicilegus*<br>*M. spretus* | -174 |
| **CRE half sequence** | ATGC | *M. cookii* | -312, -328, -404, -557 |
| | | *M. famulus* | -272, -288, -364, -472 |
| | | *M. macedonicus* | -285, -301, -357, -414, -555 |
| | | *M. m. bactrianus* | -320, -336, -392, -518 |
| | | *M. m. castaneus* | -320, -335, -391, -510 |
| | | *M. m. domesticus* | -314, -330, -386, -488 |
| | | *M. m. musculus* | -320, -335, -391, -407 |
| | | *M. pahari* | -275, -302, -318, -407, -658, -909, -1112, -1113 |
| | | *M. spicilegus* | -320, -335, -488, -560 |
| | | *M. spretus* | -320, -335, -391, -550 |
| | TTCA | *M. cookii* | -193, -210, -594, -620, -681, -951 |
| | | *M. famulus* | -193, -229, -509, -535, -596, -878 |
| | | *M. macedonicus* | -195, -208, -552, -895 |
| | | *M. m. bactrianus* | -187, -204, -217, -555, -618, -897 |
| | | *M. m. castaneus* | -187, -204, -217, -547, -610, -880 |
| | | *M. m. domesticus* | -187, -204, -217, -525, -588, -829 |
| | | *M. m. musculus* | -187, -204, -217, -555, -618, -893 |
| | | *M. pahari* | -206, -219, -426, -610, -707, -747, -808 |
| | | *M. spicilegus* | -187, -204, -217, -492, -525, -557, -798 |
| | | *M. spretus* | -180, -204, -217, -410, -612, -866 |
